# Dipotassium disulfanilamidate trihydrate

**DOI:** 10.1107/S1600536811026791

**Published:** 2011-07-09

**Authors:** Fiona N.-F How, Z. A. Rahima, Hamid Khaledi, Hapipah Mohd Ali

**Affiliations:** a Department of Biomedical Sciences, Kulliyah of Science, IIUM Kuantan, 25200 Kuantan, Malaysia; b Department of Chemistry, University of Malaya, 50603 Kuala Lumpur, Malaysia

## Abstract

The asymmetric unit of the title compound, 2K^+^·2C_6_H_7_N_2_O_2_S^−^·3H_2_O, consists of two potassium cations located on mirror planes, one sulfanilamidate anion in a general position and one and a half mol­ecules of water, one of which is also located on a mirror plane. One potassium cation is seven-coordinated by six sulfonyl O atoms and one water mol­ecule, whereas the other is surrounded by six water O atoms and two sulfonyl O atoms. In the crystal structure, the components are connected into polymeric sheets in the *bc* plane. The two-dimensional structure is consolidated by N—H⋯O, O—H⋯O, O—H⋯N and C—H⋯π inter­actions. The layers are further linked into a three-dimensional network *via* N—H⋯O, N—H⋯N and O—H⋯N hydrogen bonds.

## Related literature

For the structures of similar potassium salts, see: Gowda *et al.* (2011 ▶) and references cited therein; Moers *et al.* (2001 ▶). For the structure of sodium sulfanilamide monohydrate, see: Moreno & Alleaume (1968 ▶).
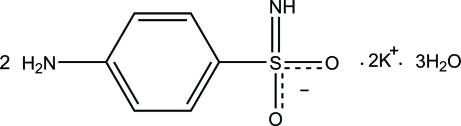


## Experimental

### 

#### Crystal data

2K^+^·2C_6_H_7_N_2_O_2_S^−^·3H_2_O
*M* *_r_* = 474.64
Orthorhombic, 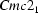

*a* = 23.8174 (4) Å
*b* = 10.9141 (2) Å
*c* = 7.4645 (1) Å
*V* = 1940.36 (5) Å^3^
*Z* = 4
Mo *K*α radiation
μ = 0.75 mm^−1^
*T* = 100 K
0.33 × 0.27 × 0.10 mm

#### Data collection

Bruker APEXII CCD diffractometer
Absorption correction: multi-scan (*SADABS*; Sheldrick, 1996 ▶) *T*_min_ = 0.791, *T*_max_ = 0.929
8594 measured reflections
2157 independent reflections
2149 reflections with *I* > 2σ(*I*)
*R*_int_ = 0.015

#### Refinement

*R*[*F* ^2^ > 2σ(*F* ^2^)] = 0.015
*wR*(*F* ^2^) = 0.042
*S* = 1.11
2157 reflections
145 parameters
7 restraints
H atoms treated by a mixture of independent and constrained refinement
Δρ_max_ = 0.26 e Å^−3^
Δρ_min_ = −0.23 e Å^−3^
Absolute structure: Flack (1983 ▶), 985 Friedel pairs
Flack parameter: 0.04 (3)

### 

Data collection: *APEX2* (Bruker, 2007 ▶); cell refinement: *SAINT* (Bruker, 2007 ▶); data reduction: *SAINT*; program(s) used to solve structure: *SHELXS97* (Sheldrick, 2008 ▶); program(s) used to refine structure: *SHELXL97* (Sheldrick, 2008 ▶); molecular graphics: *X-SEED* (Barbour, 2001 ▶); software used to prepare material for publication: *SHELXL97* and *publCIF* (Westrip, 2010 ▶).

## Supplementary Material

Crystal structure: contains datablock(s) I, global. DOI: 10.1107/S1600536811026791/om2446sup1.cif

Structure factors: contains datablock(s) I. DOI: 10.1107/S1600536811026791/om2446Isup2.hkl

Supplementary material file. DOI: 10.1107/S1600536811026791/om2446Isup3.cml

Additional supplementary materials:  crystallographic information; 3D view; checkCIF report

## Figures and Tables

**Table 1:** Hydrogen-bond geometry (Å, °) *Cg*1 is the centroid of the C1–C6 ring.

*D*—H⋯*A*	*D*—H	H⋯*A*	*D*⋯*A*	*D*—H⋯*A*
N1—H1⋯O1^i^	0.88 (1)	2.17 (1)	2.9854 (15)	154 (2)
N2—H2*A*⋯N1^ii^	0.90 (1)	2.13 (1)	3.0109 (16)	166 (1)
N2—H2*B*⋯O3^iii^	0.91 (1)	2.12 (1)	3.0183 (15)	171 (1)
O3—H3*A*⋯N2^iv^	0.85 (2)	1.99 (2)	2.8366 (15)	172 (2)
O3—H3*B*⋯O2^v^	0.83 (2)	1.95 (2)	2.7561 (13)	164 (2)
O4—H4⋯N1^vi^	0.79 (1)	2.05 (1)	2.8291 (14)	175 (2)
C2—H2⋯*Cg*1^i^	0.95	2.98	3.5872 (14)	123
C5—H5⋯*Cg*1^ii^	0.95	2.66	3.4531 (14)	141

Symmetry codes: (i) 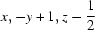; (ii) 
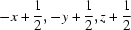
; (iii) 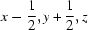; (iv) 
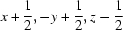
; (v) 
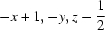
; (vi) 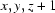.

**Fig. 1.**
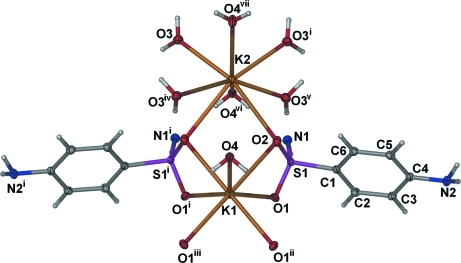
Thermal ellipsoid plot of the title compound at the 50% probability level. Hydrogen atoms are drawn as spheres of arbitrary radius. Symmetry code: i = -x + 1, y, z; ii = x, -y + 1, z + 1/2; iii = -x + 1, -y + 1, z + 1/2; iv = x, -y, z + 1/2; v = -x + 1, -y, z + 1/2; vi = x, y, z - 1; vii = -x + 1, -y, z - 1/2.

**Fig. 2.**
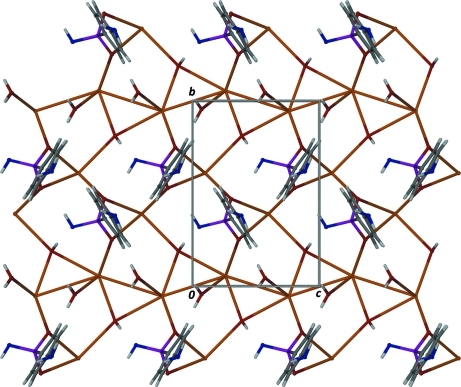
Packing view looking down the crystallographic α axis showing the two dimensional coordination polymer netwrok.

**Fig. 3.**
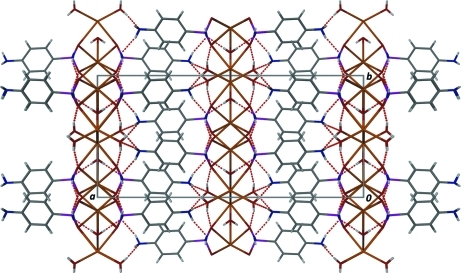
Packing view looking down the crystallographic c axis. H-bonds are shown as red dashed lines.

## supplementary crystallographic information

### Comment

The title potassium salt (Fig. 1) was obtained through the deprotonation of sulfanilamide by KOH. The structure contains two types of potassium cation, one of which, K1, is hepta-coordinated by six sulfonyl O atoms and one water molecule while the other one, K2, is octa-coordinated by six water O atoms and two sulfonyl O atoms. Both potassium atoms and one water oxygen atom, O4, are placed on a mirror plane.

The bond distance of S—N [1.5410 (12) Å] implies its double bond character and is slightly shorter than the observed values (~ 1.58 Å) in similar potassium salts (Gowda *et al.*, 2011; Moers *et al.*, 2001), but comparable with the bond length reported for sodium sulfanilamide (Moreno & Alleaume, 1968). The S1—O1 and S1—O2 distances of 1.4686 (9) and 1.4705 (9) Å attest to delocalization of the negative charge over the O—S—O fragment. These values are similar to the corresponding values in sodium sulfanilamide (1.45–1.46 Å).

In the crystal, coordination polymeric layers are formed in the *bc* plane (Fig. 2). The two dimensional structure is supplemented by N—H···O, O—H···O, O—H···N and C—H···π interactions (Table 1). The layers are further linked into a three dimensional network *via* N—H···O, N—H···N and O—H···N hydrogen bonds (Fig. 3)

### Experimental

Sulfanilamide (2 g) in ethanol (30 ml) was mixed with an equimolar amount of KOH in 90% ethanol (10 ml). The solution was then refluxed for 2 hr and left to cool down at room temperature. The colorless crystals of the the potassium salt were obtained within a day.

### Refinement

The C-bound H atoms were placed at calculated positions and were treated as riding on their parent C atoms with C—H = 0.95 Å. The N– and O-bound H atoms were located in a difference Fourier map, and refined with distance restraints of O—H = 0.84 (2) Å and N—H = 0.91 (2) Å. For all H atoms, *U*_iso_(H) was set to 1.2(1.5 for hydroxyl)*U*_eq_(carrier atom). An absolute structure was established using anomalous dispersion effects; 985 Friedel pairs were not merged.

### Crystal data

**Table:**

2K^+^·2C_6_H_7_N_2_O_2_S^−^·3H_2_O	*F*(000) = 984
*M**_r_* = 474.64	*D*_x_ = 1.625 Mg m^−^^3^
Orthorhombic, *C**m**c*2_1_	Mo *K*α radiation, λ = 0.71073 Å
Hall symbol: C 2c -2	Cell parameters from 9001 reflections
*a* = 23.8174 (4) Å	θ = 2.7–30.5°
*b* = 10.9141 (2) Å	µ = 0.75 mm^−^^1^
*c* = 7.4645 (1) Å	*T* = 100 K
*V* = 1940.36 (5) Å^3^	Blade, colorless
*Z* = 4	0.33 × 0.27 × 0.10 mm

### Data collection

**Table:**

Bruker APEXII CCD diffractometer	2157 independent reflections
Radiation source: fine-focus sealed tube	2149 reflections with *I* > 2σ(*I*)
graphite	*R*_int_ = 0.015
φ and ω scans	θ_max_ = 27.0°, θ_min_ = 2.1°
Absorption correction: multi-scan (*SADABS*; Sheldrick, 1996)	*h* = −30→30
*T*_min_ = 0.791, *T*_max_ = 0.929	*k* = −13→13
8594 measured reflections	*l* = −9→9

### Refinement

**Table:**

Refinement on *F*^2^	Secondary atom site location: difference Fourier map
Least-squares matrix: full	Hydrogen site location: inferred from neighbouring sites
*R*[*F*^2^ > 2σ(*F*^2^)] = 0.015	H atoms treated by a mixture of independent and constrained refinement
*wR*(*F*^2^) = 0.042	*w* = 1/[σ^2^(*F*_o_^2^) + (0.0201*P*)^2^ + 1.0371*P*] where *P* = (*F*_o_^2^ + 2*F*_c_^2^)/3
*S* = 1.11	(Δ/σ)_max_ < 0.001
2157 reflections	Δρ_max_ = 0.26 e Å^−^^3^
145 parameters	Δρ_min_ = −0.23 e Å^−^^3^
7 restraints	Absolute structure: Flack (1983), 985 Friedel pairs
Primary atom site location: structure-invariant direct methods	Flack parameter: 0.04 (3)

### Special details

**Table:**

Geometry. All e.s.d.'s (except the e.s.d. in the dihedral angle between two l.s. planes) are estimated using the full covariance matrix. The cell e.s.d.'s are taken into account individually in the estimation of e.s.d.'s in distances, angles and torsion angles; correlations between e.s.d.'s in cell parameters are only used when they are defined by crystal symmetry. An approximate (isotropic) treatment of cell e.s.d.'s is used for estimating e.s.d.'s involving l.s. planes.
Refinement. Refinement of *F*^2^ against ALL reflections. The weighted *R*-factor *wR* and goodness of fit *S* are based on *F*^2^, conventional *R*-factors *R* are based on *F*, with *F* set to zero for negative *F*^2^. The threshold expression of *F*^2^ > σ(*F*^2^) is used only for calculating *R*-factors(gt) *etc*. and is not relevant to the choice of reflections for refinement. *R*-factors based on *F*^2^ are statistically about twice as large as those based on *F*, and *R*- factors based on ALL data will be even larger.

### Fractional atomic coordinates and isotropic or equivalent isotropic displacement parameters (Å^2^)

**Table:**

	*x*	*y*	*z*	*U*_iso_*/*U*_eq_	
K1	0.5000	0.39021 (4)	0.59975 (5)	0.01535 (8)	
K2	0.5000	0.05477 (3)	0.26065 (6)	0.01442 (8)	
S1	0.404982 (11)	0.35192 (2)	0.25404 (4)	0.01158 (7)	
O1	0.42926 (4)	0.47094 (8)	0.30427 (12)	0.01535 (19)	
O2	0.42708 (4)	0.25122 (8)	0.36386 (12)	0.01634 (19)	
N1	0.41122 (4)	0.31915 (11)	0.05418 (15)	0.0147 (2)	
H1	0.4057 (7)	0.3869 (13)	−0.007 (2)	0.018*	
N2	0.15806 (5)	0.38097 (11)	0.38621 (15)	0.0148 (2)	
H2A	0.1423 (6)	0.3121 (13)	0.430 (2)	0.018*	
H2B	0.1402 (6)	0.4057 (14)	0.285 (2)	0.018*	
C1	0.33256 (5)	0.36379 (11)	0.30816 (17)	0.0124 (2)	
C2	0.30175 (5)	0.46150 (10)	0.23864 (18)	0.0138 (2)	
H2	0.3202	0.5243	0.1732	0.017*	
C3	0.24417 (5)	0.46705 (10)	0.26498 (19)	0.0134 (2)	
H3	0.2234	0.5342	0.2183	0.016*	
C4	0.21636 (5)	0.37450 (11)	0.35982 (16)	0.0126 (2)	
C5	0.24773 (5)	0.27905 (12)	0.43346 (18)	0.0144 (2)	
H5	0.2295	0.2175	0.5023	0.017*	
C6	0.30546 (5)	0.27342 (11)	0.40674 (17)	0.0138 (2)	
H6	0.3265	0.2075	0.4561	0.017*	
O3	0.58605 (4)	−0.04452 (9)	0.07181 (13)	0.0173 (2)	
H3A	0.6103 (6)	0.0020 (18)	0.023 (3)	0.026*	
H3B	0.5880 (8)	−0.1110 (15)	0.019 (3)	0.026*	
O4	0.5000	0.18900 (13)	0.8893 (2)	0.0207 (3)	
H4	0.4747 (6)	0.2267 (16)	0.929 (3)	0.031*	

### Atomic displacement parameters (Å^2^)

**Table:**

	*U*^11^	*U*^22^	*U*^33^	*U*^12^	*U*^13^	*U*^23^
K1	0.01301 (16)	0.01830 (18)	0.01474 (17)	0.000	0.000	−0.00331 (15)
K2	0.01360 (16)	0.01577 (17)	0.01391 (16)	0.000	0.000	−0.00172 (17)
S1	0.01032 (12)	0.01235 (13)	0.01206 (13)	−0.00085 (9)	0.00042 (13)	0.00013 (13)
O1	0.0144 (4)	0.0149 (4)	0.0168 (5)	−0.0035 (3)	0.0009 (3)	−0.0022 (3)
O2	0.0134 (4)	0.0169 (4)	0.0188 (5)	0.0009 (3)	−0.0002 (4)	0.0048 (4)
N1	0.0158 (5)	0.0161 (5)	0.0123 (5)	−0.0002 (4)	0.0024 (4)	−0.0005 (4)
N2	0.0118 (5)	0.0178 (5)	0.0148 (5)	0.0007 (4)	0.0004 (4)	0.0019 (4)
C1	0.0113 (6)	0.0142 (5)	0.0118 (5)	−0.0010 (5)	0.0009 (4)	−0.0027 (4)
C2	0.0163 (5)	0.0129 (5)	0.0120 (6)	−0.0015 (4)	0.0019 (5)	0.0017 (5)
C3	0.0157 (5)	0.0123 (5)	0.0121 (5)	0.0017 (4)	−0.0004 (5)	0.0008 (5)
C4	0.0121 (6)	0.0156 (6)	0.0102 (6)	−0.0001 (5)	0.0009 (4)	−0.0028 (5)
C5	0.0161 (6)	0.0136 (5)	0.0136 (5)	−0.0019 (5)	0.0017 (5)	0.0016 (5)
C6	0.0139 (6)	0.0134 (5)	0.0142 (6)	0.0010 (4)	−0.0007 (5)	0.0016 (5)
O3	0.0177 (4)	0.0155 (4)	0.0187 (5)	−0.0009 (3)	0.0033 (4)	−0.0010 (4)
O4	0.0135 (7)	0.0205 (7)	0.0280 (8)	0.000	0.000	−0.0097 (6)

### Geometric parameters (Å, °)

**Table:**

K1—O1^i^	2.7325 (9)	N1—H1	0.878 (14)
K1—O1^ii^	2.7325 (9)	N2—C4	1.4044 (16)
K1—O2^iii^	2.9014 (10)	N2—H2A	0.903 (13)
K1—O2	2.9014 (10)	N2—H2B	0.906 (14)
K1—O1	2.9121 (10)	C1—C6	1.3895 (17)
K1—O1^iii^	2.9121 (10)	C1—C2	1.3947 (17)
K1—O4	3.0810 (16)	C2—C3	1.3868 (17)
K2—O3	2.7133 (10)	C2—H2	0.9500
K2—O3^iii^	2.7133 (10)	C3—C4	1.4000 (17)
K2—O4^iv^	2.8285 (15)	C3—H3	0.9500
K2—O2^iii^	2.8648 (10)	C4—C5	1.3948 (18)
K2—O2	2.8648 (10)	C5—C6	1.3908 (17)
K2—O3^v^	3.0996 (10)	C5—H5	0.9500
K2—O3^vi^	3.0996 (10)	C6—H6	0.9500
K2—O4^vii^	3.1355 (17)	O3—K2^iv^	3.0996 (10)
S1—O2	1.4686 (9)	O3—H3A	0.852 (15)
S1—O1	1.4705 (9)	O3—H3B	0.828 (15)
S1—N1	1.5413 (12)	O4—K2^vi^	2.8284 (15)
S1—C1	1.7763 (13)	O4—K2^ix^	3.1355 (17)
O1—K1^viii^	2.7324 (9)	O4—H4	0.787 (13)
			
O1^i^—K1—O1^ii^	76.15 (4)	O3^vi^—K2—O4^vii^	132.79 (2)
O1^i^—K1—O2^iii^	105.06 (3)	O2—S1—O1	112.21 (6)
O1^ii^—K1—O2^iii^	176.54 (3)	O2—S1—N1	109.40 (6)
O1^i^—K1—O2	176.54 (3)	O1—S1—N1	114.44 (6)
O1^ii^—K1—O2	105.06 (3)	O2—S1—C1	106.00 (6)
O2^iii^—K1—O2	73.54 (4)	O1—S1—C1	105.03 (6)
O1^i^—K1—O1	127.75 (2)	N1—S1—C1	109.31 (6)
O1^ii^—K1—O1	84.18 (2)	S1—O1—K1^viii^	126.15 (5)
O2^iii^—K1—O1	92.57 (3)	S1—O1—K1	98.82 (4)
O2—K1—O1	49.62 (3)	K1^viii^—O1—K1	103.54 (3)
O1^i^—K1—O1^iii^	84.18 (2)	S1—O2—K2	128.84 (5)
O1^ii^—K1—O1^iii^	127.75 (2)	S1—O2—K1	99.32 (5)
O2^iii^—K1—O1^iii^	49.62 (3)	K2—O2—K1	101.04 (3)
O2—K1—O1^iii^	92.58 (3)	S1—N1—H1	106.9 (11)
O1—K1—O1^iii^	70.71 (4)	C4—N2—H2A	114.8 (10)
O1^i^—K1—O4	90.20 (3)	C4—N2—H2B	111.3 (10)
O1^ii^—K1—O4	90.20 (3)	H2A—N2—H2B	110.9 (15)
O2^iii^—K1—O4	93.04 (3)	C6—C1—C2	119.69 (11)
O2—K1—O4	93.04 (3)	C6—C1—S1	121.31 (10)
O1—K1—O4	138.32 (2)	C2—C1—S1	118.83 (10)
O1^iii^—K1—O4	138.32 (2)	C3—C2—C1	120.07 (11)
O3—K2—O3^iii^	98.12 (4)	C3—C2—H2	120.0
O3—K2—O4^iv^	78.50 (3)	C1—C2—H2	120.0
O3^iii^—K2—O4^iv^	78.50 (3)	C2—C3—C4	120.53 (11)
O3—K2—O2^iii^	88.89 (3)	C2—C3—H3	119.7
O3^iii^—K2—O2^iii^	153.71 (3)	C4—C3—H3	119.7
O4^iv^—K2—O2^iii^	127.79 (3)	C5—C4—C3	119.00 (11)
O3—K2—O2	153.71 (3)	C5—C4—N2	120.79 (11)
O3^iii^—K2—O2	88.89 (3)	C3—C4—N2	120.15 (11)
O4^iv^—K2—O2	127.78 (3)	C6—C5—C4	120.40 (12)
O2^iii^—K2—O2	74.64 (4)	C6—C5—H5	119.8
O3—K2—O3^v^	82.84 (3)	C4—C5—H5	119.8
O3^iii^—K2—O3^v^	150.97 (2)	C1—C6—C5	120.25 (11)
O4^iv^—K2—O3^v^	73.24 (3)	C1—C6—H6	119.9
O2^iii^—K2—O3^v^	54.88 (3)	C5—C6—H6	119.9
O2—K2—O3^v^	103.08 (3)	K2—O3—K2^iv^	84.51 (3)
O3—K2—O3^vi^	150.97 (2)	K2—O3—H3A	119.9 (14)
O3^iii^—K2—O3^vi^	82.84 (3)	K2^iv^—O3—H3A	98.5 (13)
O4^iv^—K2—O3^vi^	73.24 (3)	K2—O3—H3B	129.9 (13)
O2^iii^—K2—O3^vi^	103.08 (3)	K2^iv^—O3—H3B	69.3 (13)
O2—K2—O3^vi^	54.87 (3)	H3A—O3—H3B	106 (2)
O3^v^—K2—O3^vi^	82.79 (4)	K2^vi^—O4—K1	115.62 (5)
O3—K2—O4^vii^	74.17 (3)	K2^vi^—O4—K2^ix^	81.99 (4)
O3^iii^—K2—O4^vii^	74.17 (3)	K1—O4—K2^ix^	162.39 (5)
O4^iv^—K2—O4^vii^	137.70 (5)	K2^vi^—O4—H4	128.2 (13)
O2^iii^—K2—O4^vii^	83.57 (3)	K1—O4—H4	83.7 (15)
O2—K2—O4^vii^	83.57 (3)	K2^ix^—O4—H4	85.1 (16)
O3^v^—K2—O4^vii^	132.79 (2)		

Symmetry codes: (i) −*x*+1, −*y*+1, *z*+1/2; (ii) *x*, −*y*+1, *z*+1/2; (iii) −*x*+1, *y*, *z*; (iv) −*x*+1, −*y*, *z*−1/2; (v) *x*, −*y*, *z*+1/2; (vi) −*x*+1, −*y*, *z*+1/2; (vii) *x*, *y*, *z*−1; (viii) −*x*+1, −*y*+1, *z*−1/2; (ix) *x*, *y*, *z*+1.

### Hydrogen-bond geometry (Å, °)

**Table:**

Cg1 is the centroid of the C1–C6 ring.

**Table:**

*D*—H···*A*	*D*—H	H···*A*	*D*···*A*	*D*—H···*A*
N1—H1···O1^x^	0.88 (1)	2.17 (1)	2.9854 (15)	154.(2)
N2—H2A···N1^xi^	0.90 (1)	2.13 (1)	3.0109 (16)	166.(1)
N2—H2B···O3^xii^	0.91 (1)	2.12 (1)	3.0183 (15)	171.(1)
O3—H3A···N2^xiii^	0.85 (2)	1.99 (2)	2.8366 (15)	172.(2)
O3—H3B···O2^iv^	0.83 (2)	1.95 (2)	2.7561 (13)	164.(2)
O4—H4···N1^ix^	0.79 (1)	2.05 (1)	2.8291 (14)	175 (2)
C2—H2···Cg1^x^	0.95	2.98	3.5872 (14)	123
C5—H5···Cg1^xi^	0.95	2.66	3.4531 (14)	141

Symmetry codes: (x) *x*, −*y*+1, *z*−1/2; (xi) −*x*+1/2, −*y*+1/2, *z*+1/2; (xii) *x*−1/2, *y*+1/2, *z*; (xiii) *x*+1/2, −*y*+1/2, *z*−1/2; (iv) −*x*+1, −*y*, *z*−1/2; (ix) *x*, *y*, *z*+1.
